# Evaluation of Emergency Admissions Due to Animal-Related Incidents Based on Prehospital Emergency Medicine Data: A Retrospective Study

**DOI:** 10.7759/cureus.70919

**Published:** 2024-10-06

**Authors:** Ramiz Yazıcı, Murat Genç

**Affiliations:** 1 Emergency Department, Kanuni Sultan Süleyman Training and Research Hospital, Istanbul, TUR; 2 Emergency Medicine, Ankara Training and Research Hospital, Ankara, TUR

**Keywords:** animal bite, dog bites, emergency medical service, pre-hospital emergency, public health and safety

## Abstract

Introduction: Animal injuries, especially dog bites, are a major public health problem in both developed and developing countries. The majority of these injuries lead to emergency healthcare visits and may have serious consequences such as infection risk and organ injuries. There is a limited number of studies on the demographic and operational characteristics of animal-related 112 emergency visits in Turkey. In this study, we aimed to conduct research to fill this gap.

Materials and Methods: This retrospective study included animal-caused cases received by Ankara 112 Emergency Health Services Emergency Health Automation System (ASOS) between January 1, 2019 and December 31, 2023. A total of 3457 cases were analyzed. Data were evaluated using the IBM SPSS 27.0 program.

Results: There was an overall decreasing trend in animal-related emergency admissions between 2019 and 2023. Most cases were recorded during the summer season, especially in July. Male patients predominated (61.2%), with the majority of cases (81.7%) occurring in urban areas. Dog bites were the most common cause, accounting for 49.3% of cases. In the majority of cases, transportation time was below the Ministry of Health quality standards.

Conclusion: Dog bites account for the majority of animal-related emergency admissions. The increase in cases during the summer months emphasizes the importance of social awareness and preventive strategies. The findings of the study provide an important reference for improving emergency health services and developing strategies to prevent such cases.

## Introduction

Animal-caused injuries, an important public health problem, vary depending on geographical animal diversity. Animals can cause injuries by various mechanisms such as biting, stinging, crushing, pecking, and scratching. Although wild animal injuries are rarely seen, they are often more serious and have high mortality rates [1].

Whether fatal or not, animal-caused emergencies also bring a financial burden to society. Animal-caused emergency admissions include different causes ranging from simple soft tissue trauma to vital organ injuries and death. The goal in animal-caused trauma is to quickly transport the patient to the most appropriate hospital where they can receive quality, up-to-date, and advanced treatment as soon as possible. In traumas that require rapid intervention by nature, the reaction time of the 112 command and control center which is the emergency system of the country in all cases, the departure and arrival times of the ambulance, the vital interventions applied in the first contact with emergency health services and the arrival time of the case to the hospital are of vital importance [2].

Animal bites are an important public health problem for both developed and developing countries [3]. Such cases require urgent medical intervention, especially due to the risk of infection, injuries, and infectious diseases. In the United States of America, they account for approximately 1% of emergency room visits each year and cause an annual cost of 50 million dollars [4].

Dog bites constitute the majority of animal attacks and patients are usually children [5]. Injuries are first brought to emergency services and initial treatment is mostly performed in emergency services. In major injuries, a multidisciplinary approach is required and mortal outcomes may occur if effective treatment conditions are not met.

In Türkiye, 112 emergency health services respond quickly and effectively to emergencies such as animal bites, ensuring that patients receive appropriate medical care as soon as possible. However, there is limited research on the prevalence, demographic characteristics, geographical distribution, and impact on emergency medicine practice.

The aim of our study was to contribute to the literature by retrospectively examining 112 emergency applications caused by animals in terms of demographics, time of occurrence, reaction time of the 112 command and control center, ambulance departure and arrival times, arrival time of the case to the hospital, the region where the case was taken, the need and reason for transportation of the case, if any, and the type of animal that attacked. By analyzing these factors, we aim to identify patterns that may assist in optimizing emergency response times and resource allocation. Furthermore, understanding the characteristics of animal-related incidents can help in developing preventive measures and improving prehospital care protocols to enhance patient outcomes.

The findings obtained will constitute an important source in terms of improvements that can be made in the interventions of emergency health services for animal bite cases and strategies to be developed for the prevention of such cases.

## Materials and methods

This study is a retrospective observational study, analyzing animal-related emergency calls recorded by the Ankara 112 Emergency Health Services Automation System (ASOS) between January 1, 2019, and December 31, 2023. The study population consisted of all emergency calls related to animal-related incidents received within this time frame. The study sample includes cases of animal bites that met the inclusion criteria outlined below.

A total of 3,457 animal bite cases were included in the study. Cases were selected based on the availability of complete transport forms and key variable data. No specific sampling method was employed since the study aimed to include all eligible cases within the defined time period.

Inclusion criteria are animal bite incidents reported to the Ankara 112 Emergency Health Services between January 1, 2019, and December 31, 2023, and availability of complete transport forms and recorded data for the defined study variables.

Exclusion criteria are cases with incomplete or missing transport forms and cases lacking data on key study parameters (e.g., bite location, patient outcome, transport details).

The main variables analyzed in this study include Patient demographics (Age, gender, and address of the incident.), incident characteristics (type of animal involved, incident location, time of day, and season), medical response (Response time, transport decision, treatment administered on-site, and referral to hospital), outcomes (Discharge, hospital admission, or other outcomes following emergency response).

Definitions of specific terms include reach time (The duration between the call being received by 112 and the arrival of emergency services at the scene), incident location (Categorized as urban or rural based on official zoning of the location), animal type (Defined based on the description provided in the incident report).

Data analysis was performed using IBM SPSS 27.0 (Armonk, NY: IBM Corp.) statistical package program. Descriptive statistical methods (frequency, percentage, mean, standard deviation, median, IQR) as well as the Chi-Square (χ²) test were used to compare qualitative data. The conformity of the data to normal distribution was evaluated by the Kolmogorov-Smirnov test, skewness and kurtosis, and graphical methods (histogram, Q-Q Plot, Stem and Leaf, Boxplot). Independent Samples t-test and One-Way ANOVA test were used in the evaluation of quantitative data with normal distribution. In cases where there was a difference in multiple comparisons, the Tukey test and Bonferroni correction were applied. The statistical significance level was accepted as p=0.05.

## Results

The study included 3,457 patients with animal-related injuries. In terms of gender distribution, male patients were more frequently affected, comprising 2,115 cases (61.2%), while female patients accounted for 1,342 cases (38.8%). The age distribution of the patients showed a mean age of 35.7 ± 21.9 years, with a median age of 33.0 years (IQR: 17.0-53.0).

Regarding the geographical distribution of incidents, the majority occurred in urban areas, with 2,824 cases (81.7%) reported in urban settings, while 633 cases (18.3%) were recorded in rural areas. When analyzing the distribution of cases between 2019 and 2023, a downward trend in the number of cases per year was observed. While 757 cases were reported in 2019, this number decreased to 585 by 2023. The number of cases recorded was 714 in 2020, 701 in 2021, and 700 in 2022. Looking at the seasonal distribution, the highest number of cases occurred in summer (1,429 cases, 41.3%), followed by fall (843 cases, 24.4%), spring (729 cases, 21.1%), and winter (456 cases, 13.2%). In terms of monthly distribution, July had the highest number of cases (522 cases, 15.1%), followed by August (503 cases, 14.6%). The fewest cases were recorded in February (131 cases, 3.8%) and January (157 cases, 4.5%). Regarding the time intervals, the majority of cases occurred between 16:00 and 23:59 (1,609 cases, 46.5%), followed by 08:00 to 15:59 (1,476 cases, 42.7%), and 00:00 to 07:59 (372 cases, 10.8%). When examining cases during working and non-working hours, it was found that out-of-working-hours cases were more frequent (2,156 cases, 62.4%), compared to cases during working hours (1,301 cases, 37.6%).

The majority of cases were transferred to hospitals (2,777 cases, 80.3%). Among these, training and research hospitals (TRHs) were the most common destination, with 1,562 cases (56.2%) referred to them. State hospitals (SHs) received 1,038 cases (37.4%), and 168 cases (6.0%) were transferred to university hospitals. Additionally, 379 cases (11.0%) were recorded as non-transferred or refused admission. A total of 278 cases (8.0%) involved interhospital transfers. The most common reason for transfers was the need for a specialist physician (80.6%), with 224 cases transferred for this reason. The need for intensive care led to the transfer of 28 cases (10.1%), and 21 cases (7.6%) were transferred due to a lack of necessary medical equipment. Five cases (1.8%) were transferred due to a shortage of available beds. Additionally, 23 cases (0.7%) were treated on-site. In terms of the animals involved, dog-related incidents were the most common (1,706 cases, 49.3%), followed by non-venomous insect bites (1,033 cases, 29.9%), and scorpion stings (268 cases, 7.8%). Less common causes included bee stings (136 cases, 3.9%), venomous animals (94 cases, 2.7%), snake bites (76 cases, 2.2%), attacks by other mammals (67 cases, 1.9%), tick bites (57 cases, 1.6%), and other causes (20 cases, 0.6%) (Table 1).

**Table 1:** Characteristics of the participants *n/% ^#^Mean ± SD/Median (IQR) TRH: training and research hospital; SH: state hospital; SD: standard deviation; IQR: interquartile range

		n=3457	%
Year*	2019	757	21.6
	2020	714	20.7
	2021	701	20.3
	2022	700	20.2
	2023	585	16.9
Season*	Spring	729	21.1
	Summer	1429	41.3
	Fall	843	24.4
	Winter	456	13.2
Month*	January	157	4.5
	February	131	3.8
	March	179	5.2
	April	214	6.2
	May	336	9.7
	June	404	11.7
	July	522	15.1
	August	503	14.6
	September	376	10.9
	October	299	8.6
	November	168	4.9
	December	168	4.9
Hours*	00:00 - 07:59	372	10.8
	08:00 - 15:59	1476	42.7
	16:00 - 23:59	1609	46.5
Type of hour*	Working	1301	37.6
	Non-working	2156	62.4
Sex*	Female	1342	38.8
	Male	2115	61.2
Years^#^		35.7 ± 21.9	33.0 (17.0 – 53.0)
Urban/Rural*	Urban	2824	81.7
	Rural	633	18.3
Result*	Transfer to hospital	2777	80.3
Type of transferred hospital	TRH	1562	56.2
	SH	1038	37.4
	University hospital	168	6
	Private	9	0.3
	Transfer rejection	379	11
	Transfer- interhospital	278	8
	On-site intervention	23	0.7
Causing animal*	Dog	1706	49.3
	Non-poisonous insect	1033	29.9
	Scorpion	268	7.8
	Bee	136	3.9
	Poisonous Animal	94	2.7
	Snake	76	2.2
	Other mammals	67	1.9
	Tick	57	1.6
	Other	20	0.6

The median response time to arrive at the scene after the call was 576 seconds (IQR: 395.0 - 886.0). The median reaction time of the command center was 165.0 seconds (IQR: 101.0 - 278.0), while the station response time had a median of 34.0 seconds (IQR: 17.0 - 52.0).

When comparing the incidents based on the type of animal involved, a statistically significant difference was found in terms of urban/rural settings and transportation times (p<0.05). Dog attacks were more frequent in urban areas, while scorpion stings were more common in rural areas. The shortest transportation times were observed in dog attacks, while the longest times were associated with tick, scorpion, and snake bites (Table 2).

**Table 2:** Comparisons by causing animal *Chi-Square Test (n/%) ^#^One-Way ANOVA Test (Mean ± Standard Deviation)

	Causing Animal									P-value	Test
	Dog	Non-poisonous Insect (n=1033)	Scorpion	Bee	Poisonous Animal (n=94)	Snake	Other Mammals (n=67)	Tick	Other		
	(n=1706)		(n=268)	(n=136)		(n=76)		(n=57)	(n=20)		
Urban	1591 (93.3%)	756 (73.2%)	149 (55.6%)	89 (65.4%)	65 (69.1%)	59 (77.6%)	51 (76.1%)	46 (80.7%)	18 (90.0%)	<0.001*	χ^2^=361.674
Rural	115 (6.7%)	277 (26.8%)	119 (44.4%)	47 (34.6%)	29 (30.9%)	17 (22.4%)	16 (23.9%)	11 (19.3%)	2 (10.0%)		
Time to reach (seconds)	640.5± 446.3	770.3± 581.3	999.1± 851.8	745.5± 504.3	886.3± 608.9	995.7± 1386.7	707.7± 500.4	1056.4± 1463.3	650.2± 273.9	<0.001^#^	F=17000
(Call - Scene Arrival)											

When analyzing by age group, no statistically significant difference was found between the groups in terms of triage codes (p>0.05). Similarly, there was no significant difference in the reason for calls between weekdays and weekends (p>0.05). However, a statistically significant difference (p<0.05) was noted between age groups regarding time intervals, working hours, hospital transfers, and the type of animal involved. In the <18 age group, cases were less frequent between 00:00-07:59, more common during working hours, more likely to be referred to university hospitals, and more often involved dog and non-poisonous insect attacks (Table 3).

**Table 3:** Comparisons by age groups *Chi-Square Test (n / %) TRH: Training and Research Hospital; SH: State Hospital

		Age Group		P-value	Test Value
		<18	≥18		
		(n=907)	(n=2550)		
Hours	00:00 - 07:59	52 (5.7%)	320 (12.5%)	<0.001^*^	χ^2^=32.374
	08:00 - 15:59	408 (45.0%)	1068 (41.9%)		
	16:00 - 23:59	447 (49.3%)	1162 (45.6%)		
Type of Hours	Working Hours	382 (42.1%)	919 (36.0%)	0.001^*^	χ^2^=10.529
	Non-working Hours	525 (57.9%)	1631 (64.0%)		
Transferred Hospital	TRH	434 (59.1%)	1128 (55.2%)	<0.001^*^	χ^2^=80.624
	SH	211 (28.7%)	827 (40.5%)		
	University Hospitals	88 (12.0%)	80 (3.9%)		
	Private Hospital	1 (0.1%)	8 (0.4%)		
Causing Animal	Dog	502 (55.3%)	1204 (47.2%)	0.001^*^	χ^2^=26.811
	Non-poisonous insect	242 (26.7%)	791 (31.0%)		
	Scorpion	62 (6.8%)	206 (8.1%)		
	Bee	27 (3.0%)	109 (4.3%)		
	Poisonous Animal	26 (2.9%)	68 (2.7%)		
	Snake	13 (1.4%)	63 (2.5%)		
	Other mammals	10 (1.1%)	57 (2.2%)		
	Tick	18 (2.0%)	39 (1.5%)		
	Other	7 (0.8%)	13 (0.5%)		

## Discussion

In this study, we aimed to contribute to the literature by examining the demographic characteristics and the reaction times of the 112-ambulance service for the animal-caused 112 calls in Ankara province within 5 years. We think that the most important finding of our study is the animal types that differ in urban and rural areas.

There has been a general downward trend in the number of cases over the years, which may reflect the positive effects of measures and awareness-raising efforts to reduce animal attacks in the community. However, a significant increase in the number of cases was observed during the summer season. This increase can be explained by the fact that people spend more time outdoors in the summer months and the risk of contact with animals increases. Similar studies in the literature also support the effect of seasonal differences on animal attacks. In the study by Hsiao et al., it was found that insect stings were significantly higher in summer compared to other seasons [6]. Considering this increase in the summer season, preventive strategies should be developed especially during this period.

The fact that dog bites constitute the majority of animal attacks reveals the need for more studies on the animal population and dog-human interactions in Türkiye. The fact that dog attacks are common in both urban and rural areas indicates that preventive measures for such cases should be expanded. In the study conducted by Cook et al., it was concluded that dog attacks were the most common type of animal attack [7]. Similarly, Abhilash and Rao found that the most common cause was dogs and most cases were observed in the summer months [8].

It is noteworthy that scorpion stings are more common in rural areas. The fact that injuries caused by venomous animals such as scorpions are more common in rural areas requires special measures to be taken to protect individuals living in these areas. Similarly, the long intervention time for tick and snake bites emphasizes the importance of rapid intervention of emergency health services in terms of timely treatment of these cases. Bagheri et al. concluded that scorpion stings were most frequently experienced in rural areas [9].

When we looked at the gender distribution, it was observed that male patients were more common. This finding may be explained by the fact that men tend to participate in more outdoor activities and are therefore more exposed to animal attacks. However, it should be taken into consideration that female patients also constitute a significant proportion, and awareness-raising and protective measures should be taken for all segments of society. Murphy et al.’s study on interventions to animal bites revealed that the types of animals were different in the attacks to which male and female patients were exposed. While women were mostly exposed to cat bites, men were exposed to dog attacks [10].

Cases such as animal bites and stings are particularly important due to the risk of infection. Especially in dog bites, the risk of infectious diseases, especially rabies, should be taken into consideration. In this context, it is of great importance that preventive measures such as rabies vaccination are effectively implemented in cases coming to emergency services. Korkmaz et al. presented a soft tissue infection developing after a dog bite [11].

Bay et al. found that animal attacks occurred mostly in the afternoon hours [12]. In our study, we think that the fact that most of the cases occurred during off-hours is a result of the increase in human mobility in outdoor areas during these hours.

The majority of patients were transferred to hospitals. It was observed that most of the transfers between hospitals were to TRHs. It is thought that the fact that TRHs are the highest-level health institutions in the Turkish health system and that they are more competent than other hospitals in intervening in complicated cases is the reason for this result. Dal et al. also reached similar results in their study [13].

When the transportation times to the cases after the calls were examined, it was observed that they were below the urban 10 min and rural 30 min times determined in the quality standards of the Ministry of Health. This shows that prehospital health systems function with the desired efficiency [14].

Comparisons were made according to age groups. The fact that there were fewer animal attacks between 00:00 and 07:59 in the group under 18 years of age was thought to be due to the pediatric population being less likely to be in environments where they would be open to animal attacks at night. The fact that pediatric patients were referred to universities more than others suggested that university hospitals are more competent in specialized pediatric specialties. Ernst also emphasized the need for specialized care in the pediatric patient population compared to the normal population [15].

In the age group comparison according to the causative animal, it was found that the group under the age of 18 was more exposed to dog attacks. It was thought that the pediatric group was less able to protect themselves compared to adults and therefore needed health services more after dog attacks. Tam et al. found that dog attacks in the pediatric population caused more serious consequences [16].

This study has several strengths that enhance its contribution to the literature. One of the key strengths is the comprehensive nature of the dataset, which includes 3,457 cases spanning a five-year period. This large sample size increases the generalizability of the findings and allows for a thorough analysis of animal-related emergency cases. Additionally, the use of real-world data from the 112 Emergency Health Services Automation System (ASOS) provides valuable insights into the practical challenges faced in prehospital emergency care. The inclusion of diverse variables, such as demographics, response times, geographical region, and the type of animal involved, enables a multifaceted exploration of the factors influencing emergency outcomes. Another strength of the study is its focus on prehospital emergency data, which is an under-researched area. By shedding light on this critical early phase of medical intervention, the study offers insights that could help optimize emergency response strategies and resource allocation.

However, this study also has certain limitations that must be considered. The retrospective design limits control over data completeness and accuracy, with some cases excluded due to missing or incomplete records. This may introduce selection bias, potentially affecting the study's findings. Moreover, the study is geographically limited to a single region (Ankara), which could restrict the applicability of the results to other areas with different emergency response systems or animal populations. Another limitation is the lack of long-term clinical outcomes, as the study focuses only on the prehospital phase. This limits the ability to assess the overall impact of the emergency interventions on patient recovery.

## Conclusions

Our study presents an important retrospective analysis examining the demographic and operational characteristics of animal-caused cases reported to 112 Emergency Health Services between 2019 and 2023 in Ankara province. The findings provide important clues on the public health impact of animal-caused injuries and show that rapid and effective response to such incidents plays an important role in the quality of emergency health services. In this context, it is necessary to improve arrival times, especially in rural areas, increase awareness-raising activities during the summer season and take more effective measures against animal attacks, especially dog bites. The data obtained will be an important reference point for future research and policies.
